# Supplementary material for: Mechanisms governing the pioneering and redistribution capabilities of the non-classical pioneer PU.1
Source: Nat Commun. 2020 Jan 21;11:402. doi: 10.1038/s41467-019-13960-2 (PMC6972792; doi:10.1038/s41467-019-13960-2)
Supplement: Supplementary file 7 — Source data [file 41467_2019_13960_MOESM7_ESM.zip › Source_Data/Figure6/Figure6B_MotifScanOutput/ETS1_PU.1/homerResults/motif37.similar.html]

motif37

## Information for motif37

A
G
C
T
A
T
G
C
C
T
G
A
G
A
T
C
C
T
A
G
A
G
C
T
A
T
C
G
C
T
G
A
A
G
T
C
T
C
A
G
  
Reverse Opposite:  

A
G
T
C
C
T
A
G
A
G
C
T
A
T
G
C
C
T
G
A
G
A
T
C
C
T
A
G
G
A
C
T
T
A
C
G
T
C
G
A
  

|  |  |
| --- | --- |
| p-value: | 1e-36 |
| log p-value: | -8.469e+01 |
| Information Content per bp: | 1.667 |
| Number of Target Sequences with motif | 611.0 |
| Percentage of Target Sequences with motif | 3.86% |
| Number of Background Sequences with motif | 595.6 |
| Percentage of Background Sequences with motif | 1.86% |
| Average Position of motif in Targets | 103.9 +/- 55.2bp |
| Average Position of motif in Background | 98.5 +/- 56.7bp |
| Strand Bias (log2 ratio + to - strand density) | 0.1 |
| Multiplicity (# of sites on avg that occur together) | 1.06 |
| Motif File: | file (matrix) reverse opposite |

### Similar de novo motifs found

|  |  |  |  |  |  |  |  |
| --- | --- | --- | --- | --- | --- | --- | --- |
| Rank | Match Score | Redundant Motif | P-value | log P-value | % of Targets | % of Background | Motif file |
| 1 | 0.838 | C T G A A G T C C T A G A G C T A T G C C T G A G A C T | 1e-33 | -77.088405 | 6.58% | 3.99% | motif file (matrix) |
| 2 | 0.861 | T C A G A G C T A T G C G T C A G A T C C T A G A C G T A C T G T C G A A G T C A C T G T A C G | 1e-31 | -72.725861 | 2.78% | 1.23% | motif file (matrix) |
| 3 | 0.858 | T A G C C T A G G A C T T A G C T C G A G A T C A C T G G A C T | 1e-31 | -71.392823 | 8.58% | 5.70% | motif file (matrix) |
| 4 | 0.849 | T C A G A G C T A T G C T C G A A G T C T C A G A C G T T A C G T C G A A G T C A C T G | 1e-28 | -65.169362 | 1.98% | 0.77% | motif file (matrix) |
| 5 | 0.804 | C T G A C G A T C A T G C G T A A G T C C T A G A G C T G T A C G T C A | 1e-28 | -65.138655 | 1.75% | 0.63% | motif file (matrix) |
| 6 | 0.870 | A G C T A T G C C G T A A G T C T C A G A G C T A T C G C T G A A T G C | 1e-26 | -61.339781 | 4.17% | 2.35% | motif file (matrix) |
| 7 | 0.838 | T C A G A C G T A T C G T C G A A G T C T A C G A G C T A G T C G T C A G A T C T A G C | 1e-16 | -38.655170 | 0.77% | 0.23% | motif file (matrix) |
